# Febrile seizures and convulsions with mild gastroenteritis: age-dependent acute symptomatic seizures

**DOI:** 10.3389/fped.2023.1151770

**Published:** 2023-07-18

**Authors:** Alberto M. Cappellari, Stefano Mariani, Gaia Bruschi

**Affiliations:** ^1^ Department of Neuroscience and Mental Health, Fondazione IRCCS Cà Granda Ospedale Maggiore Policlinico, Milan, Italy; ^2^ Department of Medical-Surgical and Transplant Pathophysiology, University of Milan, Milan, Italy

**Keywords:** febrile seizures, benign convulsions in children with mild gastroenteritis, epilepsy, electroencephalography, PPMD

## Abstract

**Background:**

Febrile seizures (FS) and benign convulsions in children with mild gastroenteritis (CwG) are acute symptomatic seizures, transiently occurring in infants and young children, probably related to the immaturity of the brain. Our paper aims to review the literature data on patients with FS and CwG.

**Methods:**

A review of series of patients with FS and CwG was performed by literature search on PubMed January 1960 to October 2022. Several parameters were considered, including epidemiology, pathophysiology, clinical features, electroencephalographic findings and other diagnostic studies, and treatment.

**Results:**

FS and CwG share an age-dependent course, but they show significant differences in the pathophysiology, clinical features, diagnostic studies, and treatment.

**Conclusion:**

Acute symptomatic seizures include seizures that are caused by acute structural brain pathologies, such as stroke, as well as seizures that are provoked by a reversible factor, such as hyponatraemia, although the two groups should be not equated. Furthermore, FS and CwG should be set apart as “age-dependent acute symptomatic seizures”, reinforcing the concept of their self-limited course over a certain period.

## 1. Introduction

Febrile Seizures (FS) and benign convulsions in children with mild gastroenteritis (CwG) are quite common in infants and young children. The American Academy of Pediatrics (AAP) defines FS as “a seizure accompanied by fever (temperature 100.4°F or 38°C by any method), without central nervous system (CNS) infection, that occurs in infants and children 6 through 60 months of age” (1). Benign convulsions associated with mild gastroenteritis (CwG) have been defined as afebrile seizures occurring in otherwise healthy children with mild acute gastroenteritis, who do not have central nervous system infection, dehydration, or electrolyte imbalances (2). However, in clinical practice, the occurrence of febrile CwG is not uncommon (3). Although the presence of fever seems to influence the clinical characteristics of seizures associated with mild gastroenteritis (4), distinguishing between febrile CwG and FS is not easy for the clinician (5).

FS and CwG are considered situation-related seizures, which are essentially transient and functional disorders of infants and young children, probably related to the immaturity of the brain (6–10). Nevertheless, the two entities show significant differences in clinical features and management (6, 11–15). Our paper aims to review the epidemiology, clinical features, diagnostic investigations and treatment options in patients with FS and CwG.

## 2. Literature search

A review of a series of patients with FS and CwG was performed by literature search on PubMed from January 1960 to October 2022. This review is not meant to be exhaustive. We have selected some of the more comprehensive articles on FS, febrile CwG and afebrile CwG. We do not pretend to have included all the better contributions, but most of those included have extensive references that should be consulted for more complete coverage. Our search terms included “Febrile seizures,” “Benign convulsions”, “Gastroenteritis”, and “Acute symptomatic seizures”. The main data are summarized in Table 1.

**Table 1:** Summary of literature data on patients with febrile seizures, afebrile convulsions associated with mild gastroenteritis and febrile convulsions associated with mild gastroenteritis. Only studies including more than 30 patients have been included.

	Lloyd et al. 2010 (16)	Zifman et al. 2011 (17)	Lee and Chung 2013 (18)	Kang et al. 2013 (19)	Hu et al. 2017 (20)	Higuchi et al. 2017 (5)	Wu et al. 2020 (4)	Cappellari et al. 2022 (3)
Study design	Retrospective	Retrospective	Retrospective	Retrospective	Retrospective	Prospective	Retrospective	Retrospective
Case number	34	44	59	59	108	126	71	294
CwG: FS	NA	NA	NA	NA	NA	NA	NA	63:231
aCwG: fCwG	23:11	18:26	27:32	42:17	59:49	76:50	41:30	NA
Gender (female predominant)	NS	NA	NS	aCwG > fCwG	aCwG > fCwG	NS	aCwG > fCwG	NS
Seizure burden	NA	NA	aCwG > fCwG	aCwG > fCwG	NA	NA	aCwG > fCwG	NA
Clustered seizures	NA	NA	aCwG > fCwG	aCwG > fCwG	aCwG > fCwG	aCwG > fCwG	aCwG > fCwG	fCwG > FS
Focal seizure predominant	NS	NA	aCwG > fCwG	aCwG > fCwG	NA	NS	aCwG > fCwG	fCwG > FS
Prolonged seizures (≥5 min)	NS	NS	fCwG > aCwG	NS	NA	NS	aCwG > fCwG	NA
Personal history of FS	NA	NS	fCwG > aCwG	fCwG > aCwG	NA	fCwG > aCwG	NS	NS
Family history of FS or epilepsy	NA	NA	NS	NS	NA	fCwG > aCwG	NS	NS
Interval between AGE onset and first seizures	NA	NA	aCwG > fCwG	aCwG > fCwG	aCwG > fCwG	aCwG > fCwG	aCwG > fCwG	NA
EEG findings	NS	NS	aCwG > fCwG	NS	NA	NS	aCwG > fCwG	NA
Neuroimaging abnormalities	aCwG > fCwG	NA	NS	NS	NA	NS	NS	NA
Pathogen identification by stool specimens, in CwG patients	Rotavirus	Rotavirus	Rotavirus and Norovirus	Rotavirus	Norovirus	Rotavirus, Norovirus, Adenovirus	Rotavirus, Adenovirus, Norovirus and Salmonella C	NA

FS, febrile seizures; aCwG, afebrile convulsions associated with mild gastroenteritis; fCwG, febrile convulsions associated with mild gastroenteritis; NA, not available; NS, not significant; EEG, electroencephalogram; AGE, acute gastroenteritis.

## 3. Epidemiology

Both FS and CwG are transient and functional disorders occurring in infants and young children (6).

### 3.1. FS

FS affects 2%–5% of children under five years of age (21), with a peak incidence around 18 months (22). Viral infections are the most frequent cause of febrile illnesses associated with FS (up to 82% of cases) (23). Some vaccinations have also been linked to FS, with the risk period following vaccination varying between vaccines (24). Other risk factors for FS include fever above 38°C, shorter fever duration and a positive family history of FS or epilepsy (25, 26). Up to 41% of children who have febrile status epilepticus (FSE) go on to experience another FS in the future, increasing their risk of other negative outcomes (27).

### 3.2. CwG

CwG occur in children aged one month to 6 years, with a peak incidence at 1–2 years of age (28). CwG mostly occur during the winter and early spring months, which is the period of the year associated with the largest circulation of several viruses such as rotavirus, norovirus and adenovirus (28–31). Furthermore, CwG have been more frequently reported in East Asian countries, suggesting that the genetic characteristics of the host may contribute to the occurrence of seizures in children with gastroenteritis (28, 32).

## 4. Pathophysiology

The rapid development of the immature CNS in childhood may increase the susceptibility to seizures in infants and young children with FS and CwG (6).

### 4.1. FS

The height of the body temperature is considered to play a major role in the pathogenesis of FS, compared to the rapidity of its rise (33, 34). Increasing the temperature of the brain has been suggested to increase neuronal firing, which in turn increases the likelihood of synchronised neuronal activity that leads to seizure induction (35). The seizure threshold varies between patients. A lower seizure threshold to excitatory input both *in vivo* (kainic acid) and *in vitro* (electrical stimulation) had been associated with prolonged FS (36). Children prone to FS produce more pro-inflammatory cytokines in the CNS, such as IL-1Beta, which might induce seizures (37). Also, the type of infection seems to play a role in the pathogenesis of FS, with human herpes virus 6 (HHV6), viral upper respiratory tract infections and gastroenteritis frequently involved.

### 4.2. CwG

Among the various organisms causing gastroenteritis, rotavirus has been detected in a wide number of stool specimens of CwG patients (28, 38–41). There are different hypotheses on the relationship between rotavirus infection and its effect on the CNS. One hypothesis is that rotavirus may directly invade the CNS via the bloodstream (42, 43). Indeed, rotavirus RNA in the cerebrospinal fluid (CSF) has been reported in patients with gastroenteritis and seizures (43, 44). Another hypothesis is that rotavirus may indirectly provoke seizures by various mediators produced in the gastrointestinal tract and released in the general circulation (45).

## 5. Clinical features

FS and CwG are benign conditions sharing several clinical and prognostic features (6).

### 5.1. FS

Children with FS can develop seizures within one hour of the onset of the fever (21% of cases), between 1 and 24 h of the fever (57%) or more than 24 h after the fever (22%) (46). However, the fever can occur at any time, not infrequently after the seizure. Children with FS have higher temperatures with illness compared with febrile controls (47). Seizure types include tonic-clonic seizures, which can be asymmetrical, or focal impaired awareness seizures. FS have been classified as simple FS or complex based on duration, recurrence, and presence of focal features (48). Febrile seizures have an average duration of 4–7 min, with only 10%–15% of them lasting longer than 10 min (49). Simple FS are generalized seizures, with duration <15 min, and not recurrent within 24 h. Complex FS are seizures either focal, with a duration >15 min, or repetitive within 24 h. Most FS are simple FS, while 25%–35% are complex FS (50).

Up to a third of children with FS have a recurrence, and 75% of these occur within one year (51). Risk factors for recurrent FS include age at onset <18 months, history of FS in a first-degree relative, low grade of fever associated with seizures (<39°C), short duration of fever before a seizure (<1 h) and multiple seizures during the same febrile illness (50). EEG findings have also been reported as an independent risk factor for FS recurrence (50).

### 5.2. CwG

In many cases of CwG, seizures follow gastrointestinal symptoms, although they can also occur before or simultaneously with the development of diarrhoea (41).

Although the seizures are mostly reported as generalized tonic-clonic, ictal EEG recordings have always demonstrated a focal origin (41, 52). Seizures are mostly brief (<5 min), and often repetitive, usually occurring in clusters ranging from one to 8 seizures within a 24 h period (19). Cluster seizures were observed in 13%–75% of patients with CwG (53).

The overall prognosis of CwG is favourable, and the incidence of seizure recurrence is low (54). The risk of recurrence can be predicted by age at onset <18 months and repeated seizures over 24 h (54).

## 6. Electroencephalographic findings

EEG could play a role in predicting FS recurrence (50) or epilepsy development (55) in patients with FS, while the role of EEG is of limited value in the evaluation of CwG since most EEG findings return to normal during the follow-up period (41).

### 6.1. FS

There is a longstanding debate on the usefulness of EEG in children with FS (56). The value of EEG in patients with simple FS has been traditionally denied (1), although some recent papers suggest its usefulness. If an EEG is obtained, it should be taken at least 48 h after the FS to prevent conflating postictal electrical activity with aberrant activity (21). First, pseudo-petit mal discharge (PPMD) pattern and abnormal EEG have been recently reported as independent risk factors for FS recurrence (50). Second, PPMD has been reported as a marker of favourable prognosis in terms of epilepsy development, since epilepsy has been diagnosed in patients without PPMD but not in those with PPMD (55). The value of EEG in patients with complex FS remains controversial (57), and the absence of epileptiform activity does not exclude seizures (58).

### 6.2. CwG

Interictal EEG findings have been usually reported as normal in CwG, although some patients may initially present slow background activity, focal spikes or epileptiform discharges. Ictal EEG recordings reveal a focal onset, usually with secondary generalization and rarely with persisting focality (9, 41, 59).

## 7. Other diagnostic studies

The role of diagnostic studies is quite different in FS and CwG.

### 7.1. FS

Blood tests are not necessary in the presence of typical history and physical examination (60). In patients with simple FS, a lumbar puncture should be performed in children with a history and clinical features of meningitis, while it is an option in infants between 6 and 12 months of age with incomplete or unknown immunization status, as well as in a child pretreated with antibiotics (1). A lumbar puncture should also be considered in children with complex FS (60). Neuroimaging studies, including cranial computed tomography (CT) and magnetic resonance imaging (MRI), are not routinary indicated in children with FS (60–62). Imaging studies should be performed in patients with focal neurological findings, raised intracranial pressure or suspected intracranial structural pathology (60, 63, 64).

### 7.2. CwG

Diagnosis of CwG is based on clinical findings. Laboratory investigations and neuroimaging are not necessary, except in a few selected cases (65). Reversible abnormalities of high intense signal of the splenium of the corpus callosum on brain MRI have been reported in patients with CwG associated with rotavirus infection (66).

## 8. Treatment

FS and CwG have significant differences in management (6).

### 8.1. FS

Most FS are self-limited and stop on their own before patients arrive at the hospital. However, seizures lasting longer than 5 min are unlikely to stop, and a benzodiazepine is required to break the seizure (67). Therefore, intervention to stop the seizure usually is unnecessary in simple FS, since the seizure has typically resolved by the time the child is evaluated by a physician, while the treatment may be required in complex FS if the seizure is still ongoing by the time the child arrives at a medical facility (68). Intravenous lorazepam and diazepam have been reported to have similar rates of seizure resolution and respiratory depression. When intravenous access is unavailable, buccal midazolam or rectal diazepam are good alternatives (69).

Given the usually benign course of FS and the risk of adverse effects with medications, there is currently no role for prophylactic anti-seizure drugs in preventing FS. The use of antipyretics does not decrease the risk of FS (69).

### 8.2. CwG

Antiseizure drugs are not necessary if the patient has seizures of brief duration, even if they occur in clusters. Prolonged seizures may respond to conventional therapy, although some authors report that benzodiazepines were effective in stopping the seizures in only a limited number of cases (38%) (28, 41, 70, 71). Some authors suggest that carbamazepine is the most effective drug at the dose of 5 mg/Kg/die to treat prolonged seizures. Although there is no consensus on the drug of choice for CwG treatment, all authors agree to avoid benzodiazepines in the routinary treatment of patients with CwG (28).

## 9. Conclusion

FS and CwG are considered situation-related seizures, occurring in infants and young children (6–10). In clinical practice, several terms, such as “situation-related seizure”, “provoked seizure”, and “reactive seizure” are frequently used, but the ILAE has proposed that these terms are synonymous with and should be replaced by the term “acute symptomatic seizure” (72). Acute symptomatic seizures have been defined as seizures that occur within a certain period of an inciting event (73). A major issue in the definition of acute symptomatic seizures is the difficulty in combining, in a single concept, both seizures that are caused by acute structural brain pathologies, such as stroke, and seizures that are provoked by a reversible factor such as hyponatraemia, which should be not equated (74). In our opinion, FS and CwG should not be equalled to other symptomatic seizures too, owing to their propensity to exclusively occur on the immature brain. Indeed, FS and CwG could be set apart as “age-dependent acute symptomatic seizures”, reinforcing the concept of their self-limited course over a certain period. This term is not so far from other terms used to indicate specific age-dependent neurologic disorders, such as “self-limited epilepsies” (75) or transient benign paroxysmal movement disorders in infancy (76). Although the theoretical concept and definition of an acute symptomatic seizure suggests that the seizure recurrence risk should be relatively low, the risk varies according to the causes of acute symptomatic seizures (77). Among the age-dependent acute symptomatic seizures, the prognosis for subsequent epileptic seizures in children with simple FS is similar to that of the general population, while the risk of afebrile seizures is increased to 15%–20% among children with complex FS (78).

## 10. Open issues

There is controversy regarding whether benign seizures occurring in gastroenteritis with fever should be placed in the category of CwG (6). Seizures occurring in gastroenteritis with fever have been regarded as “FS”, while those occurring during gastroenteritis without fever are classified as “CwG”. However, febrile seizures during viral gastroenteritis may clinically resemble those of CwG rather than those of FS concerning the frequency of clustered seizures and the antiepileptic drug responses, suggesting that they may have a pathogenic mechanism distinct from FS due to other causes (6). Furthermore, we recently reported that children with febrile CwG showed a higher rate of complex seizures as compared with those with FS (3). Overall, these findings suggest that the relationship among FS, febrile CwG and afebrile CwG is quite complex. Although there are some studies investigating the difference between febrile CwG and FS, they are few single-center studies with a small number of patients (5). Future studies and discussions are required to resolve this issue.
